# Low-Diffusion Fricke Gel Dosimeters with Core-Shell Structure Based on Spatial Confinement

**DOI:** 10.3390/ma14143932

**Published:** 2021-07-14

**Authors:** Wei Zhang, Kaikai Wang, Yufeng Zeng, Xiaodan Hu, Xiaohong Zhang, Shuquan Chang, Haiqian Zhang

**Affiliations:** 1 College of Materials Science and Technology, Nanjing University of Aeronautics and Astronautics, Nanjing 211100, China; zhangwei_nuaa@outlook.com (W.Z.); wkk_nuaa@163.com (K.W.); zengyufeng@163.com (Y.Z.); huxiaodan@nuaa.edu.cn (X.H.); zhangxiaohong@nuaa.edu.cn (X.Z.); 2 Jiangsu Key Laboratory for Biomaterials and Devices, Southeast University, Nanjing 210096, China

**Keywords:** Fricke gel dosimeter, ion diffusion, coating, spatial confinement, PDMS

## Abstract

The diffusion of ferric ions is an important challenge to limit the application of Fricke gel dosimeters in accurate three-dimensional dose verification of modern radiotherapy. In this work, low-diffusion Fricke gel dosimeters, with a core-shell structure based on spatial confinement, were constructed by utilizing microdroplet ultrarapid freezing and coating technology. Polydimethylsiloxane (PDMS), with its excellent hydrophobicity, was coated on the surface of the pellets. The concentration gradient of the ferric ion was realized through shielding half of a Co-60 photon beam field size, and ion diffusion was measured by both ultraviolet-visible spectrophotometry and magnetic resonance imaging. No diffusion occurred between the core-shell pellets, even at 96 h after irradiation, and the diffusion length at the irradiation boundary was limited to the diameter (2–3 mm) of the pellets. Furthermore, Monte Carlo calculations were conducted to study dosimetric properties of the core-shell dosimeter, which indicated that a PDMS shell hardly affected the performance of the dosimeter.

## 1. Introduction

Modern radiation therapy technologies, including intensity modulated radiation therapy and stereotactic radiotherapy, play a critical role for tumor treatment [1]. The aim of personalized therapy is to maximize the dose to the cancerous tissue, while minimizing the dose that is delivered to the healthy tissue [2]. As a result, there is a steep gradient in the three-dimensional (3D) radiation dose field. Determining the dose distribution accurately is vital for local tumor control [3,4]; however, dosimetry measurement is presently realized by the ionization chamber and the film dosimeter. It is challenging to accurately and rapidly map the complex 3D dose [5].

3D dosimetry systems are proposed to address the abovementioned issues. These systems mainly include Fricke gels [6,7,8,9], polymer gels [10,11,12], radiochromic gels and plastics [13,14,15,16]. Fricke gels have been developed over the years and are good candidates for 3D dose mapping due to their excellent tissue equivalence, simple preparation and no oxygen effect. However, the ferrous ions (Fe^2+^) in Fricke gels are oxidized to ferric ions (Fe^3+^) upon irradiation, which leads to the poor stability of spatial dose information storage, owing to diffusion of Fe^3+^ [17]. Many research studies have focused on reducing ion diffusion in Fricke gels, including optimization of gel matrix materials [18,19,20], design of Fe^3+^ selective ligand [21,22,23,24], improvement of cross-linking method for gels [25,26], and addition of nano-sized clay particles [27,28]. In addition, strategies based on spatial confinement to suppress diffusion have also been proposed. Aparecida et al. designed a polyethylene (PE) phantom with hexagonal ‘‘honeycomb’’ cells filled with Fricke gel [29], and the dose distribution maps proved that a honeycomb-like structure prevents ion diffusion from one to another. However, the large size of the cells (5 mm) and the walls (~0.8 mm) was not beneficial to high-resolution dose measurement. Besides, the internal honeycomb cells would be not applicable for making complex phantoms. Based on the confined interfacial modification strategy, Yao et al. [30] succeeded in limiting the diffusion between hydrogels at the macro-scale and molecular scale through the construction of superhydrophobic diffusion barriers. Recently, our group [31] assembled W_1_/O/W_2_ multiple emulsions coated with Fricke hydrogel, where diffusion coefficient of Fe^3+^ was reduced to 0.17 mm^2^/h. Hydrophobic coatings were also significantly beneficial in suppressing diffusion [32,33], however, it was largely ignored in the study of Fricke gel dosimeters.

Here, the hydrophobic polydimethylsiloxane (PDMS), used as diffusion barriers, were coated on the surface of Fricke-PVA-xylenol orange (FPX) pellets prepared by microdroplet and ultrarapid freezing technology; then a novel Fricke gel dosimeter with core-shell structure (FPX@PDMS), based on spatial confinement, was constructed accordingly. PDMS coatings with excellent mechanical property and optical transparency effectively prevented the deformation of FPX pellets and ensured the accuracy of optical measurements. The diffusion between core-shell FPX@PDMS pellets was reduced to zero, and Fe^3+^ diffusion within FPX pellets was also very low. Importantly, PDMS coatings did not affect the dosimetry at the MeV energy levels, which indicated its potential in practical applications.

## 2. Materials and Methods

### 2.1. Materials and Chemicals

Poly(vinyl alcohol) (PVA) with a molecular weight of 105,000 (guaranteed reagent grade) was obtained from Sinopharm Chemical Reagent Co., Ltd. (Shanghai, China); Fe(NH_4_)_2_(SO_4_)_2_·6H_2_O, H_2_SO_4_ (98%) and xylenol orange (XO) were of analytical grade from Aladdin Industrial Corporation (Shanghai, China); liquid PDMS (Sylgard 184) was purchased from Dow Corning Corporation (Midland, MI, USA). None of the chemicals were subjected to any further purification. Ultrapure water (resistivity = 18.25 MΩ cm) by Milli-Q system was used through whole process, without special illustration. Liquid nitrogen was purchased from Shangyuan Industrial Gas Plant (Nanjing, China).

### 2.2. Preparation of FPX Precursor Solution

In a typical procedure, the Fricke solution was prepared according to the following compositions: XO, 0.1 mM; Fe(NH_4_)_2_(SO_4_)_2_·6H_2_O, 0.1 mM; and H_2_SO_4_, 25 mM. Aqueous PVA solution (10%, *w*/*w*) was prepared by dissolving PVA powder in aqueous H_2_SO_4_ (25 mM) at 90 °C, and the mixture was stirred vigorously for 3 h to obtain a homogeneous and clear solution, and then cooled to room temperature. In order to ensure no water-loss, a reflux condenser was used during preparation. The FPX precursor solution was obtained by uniformly mixing the Fricke solution with PVA solution (10%, *w*/*w*) in a ratio of 1:5 (*v*/*v*); it was left to stand for a while at room temperature in the dark to remove any bubbles before subsequent operations.

### 2.3. Preparation and Fabrication of Hydrogel Pellets

#### 2.3.1. Preparation of FPX Hydrogel Pellets

**Figure 1:**
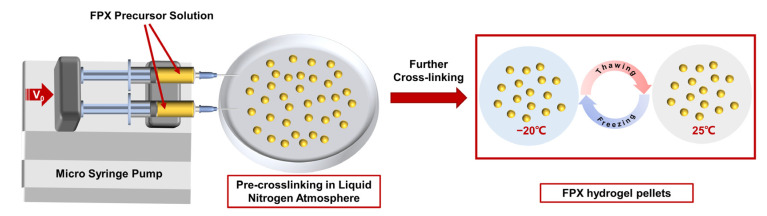
Schematic diagram of preparation process of FPX hydrogel pellets.

The preparation process of FPX hydrogel pellets was shown in Figure 1. The homemade apparatus mainly consisted of two parts: the pellet generator and collector. The former included a dual-channel high-precision syringe pump and two 10 mL syringes with needles of an inner diameter of 0.8 mm filled with FPX precursor solution, and the injection rate could be precisely tuned to achieve the high-throughput preparation of high-quality FPX hydrogel pellets. The latter consisted of a 9-cm-diameter petri dish filled with enough liquid nitrogen and a micro-oscillator (75-2A, Shanghai Medical Analytical Instrument Factory, Shanghai, China) to prevent the adhesion between pellets and ensure monodispersity. The vertical distance between syringe needles and the petri dish was fixed at 10 cm.

The FPX precursor solution was firstly squeezed out from the needles and became FPX droplets, and the droplets were secondly pre-crosslinked into pellets under an extremely cold (−196 °C) liquid nitrogen atmosphere, then these primary pellets were repetitively frozen (−20 °C, 2 h) and thawed (25 °C) for three times [10] to obtain the ultimate FPX hydrogel pellets with excellent mechanical strength.

#### 2.3.2. Fabrication of Core-Shell FPX@PDMS Pellets

The fabrication of core-shell FPX@PDMS pellets was carried out according to the following procedure: firstly, a two-component PDMS was used, where the base and curing agent were mixed at a ratio of 10:1 (*w*/*w*). To remove the bubbles caused by thorough mixing, the mixture was degassed in vacuum. Afterwards, it was poured in a petri dish and heated at 50 °C for 70 min in order to reach a semi-cured state, and it was then was cooled down to room temperature. Afterwards, FPX pellets were immersed in the semi-cured PDMS and were then carefully pulled out. Owing to the high viscosity (3500 mPa.s) of PDMS prepolymer, the surface of the FPX pellets could be easily coated with a thin layer of PDMS. Finally, core-shell FPX@PDMS pellets were obtained once the PDMS had been completely cured at room temperature.

### 2.4. Preparation of 3D FPX@PDMS in PVA Substrate Dosimeter

To demonstrate the potential of FPX@PDMS pellets in gel dosimeter, the construction of a 3D dosimeter is necessary. As proof of this concept, an FPX@PDMS pellets array (6 × 5) dosimeter in PVA hydrogel substrate was prepared. First of all, the PVA solution (10%, *w*/*w*) was poured into a cuboid mold. Secondly, the as-prepared FPX@PDMS pellets were immersed in the PVA substrate in an ordered alignment. Following this, it was repeated frozen and thawed to make the PVA hydrogel fully crosslinked. Finally, once the whole gel had demolded, the 3D FPX@PDMS dosimeter was constructed. As a control, the conventional 3D Fricke gel dosimeter was also prepared when the same procedure was applied to FPX precursor solution, except the immersion of FPX@PDMS pellets.

### 2.5. Characterization of FPX Pellets, FPX@PDMS Pellets and PDMS Coatings

**Figure 2:**
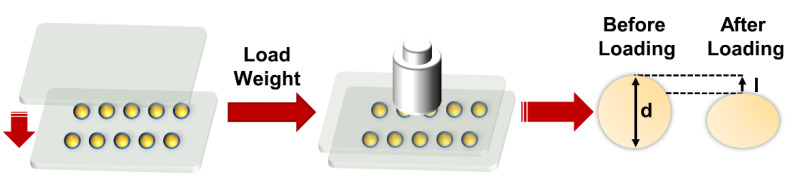
Schematic diagram of compression properties characterization of pellets.

All the optical images of pellets and coatings morphology were recorded by a digital camera (S9800, FinePix, Tokyo, Japan). The sizes were measured by a high-precision (±0.02 mm) vernier caliper (CD-P15S, Mitutoyo, Kawasaki, Japan), and the size distribution of FPX pellets was obtained by measuring the diameter of fifty pellets. The schematic diagram of compression properties characterization of the pellets was shown in Figure 2; “d” was the initial diameter of pellets, and “l” was the diameter change of the pellets after being compressed. Ten FPX@PDMS pellets were evenly distributed in the center position between two glass slides, and the weights were loaded on the surface of the slide as the pressure on the pellets and the deformation (defined as l/d) of the pellets as a function of pressure was recorded. The role of PDMS coatings in suppressing diffusion was characterized by monitoring the release of XO in the FPX pellets. FPX pellets and FPX@PDMS pellets were immersed in 100 mL of ultrapure water, respectively, under continuous stirring in order to create a steep concentration gradient; then 5 mL solution was taken out to measure the absorbance at 435 nm which was the absorption peak of XO at different time points (UV-2550, Shimadzu, Kyoto, Japan), and meanwhile the same volume of ultrapure water was added back.

### 2.6. Rheological Test of PVA Hydrogels Support

A dynamic rheometer (DHR-2, Milford, CT., USA), with a parallel plate geometry, was used for frequency sweep to characterize the PVA gels support. The absolute strain range was set to 0.5%, and the angular frequency (ω) varied from 1 to 100 rad/s.

### 2.7. Mechanical Strain Analysis at the Micro-Level

The micro-mechanical properties of FPX and FPX@PDMS pellets were detected by an atomic force microscopy (AFM) (MFP-3D, Oxford Instruments Asylum Research, Oxford, UK) [34]. During the measurements, the AFM worked in AC tapping mode to image the microscopic morphologies of the samples. The silicon nitride cantilever with a spring constant 0.1 N/m, which was calibrated based on the equipartition theorem, was used to acquire the force spectroscopy in the closed-loop mode. All measurements were conducted in air, at 25 °C. Young’s modulus was calculated according to Hertz model [35]:

$$F=\frac{4ER^{1/2}\delta^{3/2}}{3\left(1-\nu^{2}\right)}$$

where *F* and *E* is the force and Young’s modulus, respectively. *R* is the radius of the AFM tip and *ν* is Poisson’s ration which were 7 nm and 0.5, respectively. *δ* is the indentation.

### 2.8. Diffusion Measurements

The FPX precursor solution was poured into the cuvette with an optical path of 1 cm, then it was sealed with the Parafilm and repetitively frozen and thawed three times to obtain the traditional FPX dosimeter. To determine the diffusion rate of Fe^3+^ in gel, a steep dose field in dosimeters was required. Diffusion measurements were performed on the as-prepared gel by applying gamma irradiation (^60^Co source provided by Nanjing University of Aeronautics & Astronautics, Nanjing, China) to the top half of the dosimeters. By contrast, the bottom half was shielded from irradiation by using a 30 cm lead brick. A total dose of 30 Gy, calibrated by an ionization chamber (PTW 30013, Freiburg, Germany), was delivered to the irradiated area at room temperature, which could completely oxidize Fe^2+^ to Fe^3+^ to create a steep gradient [36]. It should be noted that the diffusion coefficient inside the pellets is the same as that of traditional a FPX dosimeter, and this is because the diffusion coefficient only depends on the compositions of the material and not on the specific shape.

To quantitatively monitor the diffusion process, absorbances at 585 nm (absorption peak of Fe^3+^-XO complex) [36] of 0, 5, 10, 15, 20 mm positions were measured (UV-2550, Shimadzu, Kyoto, Japan); where the interface at the irradiated area and unirradiated area corresponded to the 10 mm position, and the axis is positive in the vertical downward direction of the cuvette, the absorbance as a function of time was recorded. Particularly, the commercial spectrophotometer was partially modified in order to ensure the accurate absorbance measurement at different positions. A mask was first added at the entrance of the beam into the cuvette to ensure as much narrow beam incidence as possible and confine the measurements to the specific narrow regions. Then, a height-adjustable cuvette holder with scale lines was customized to replace the usual height-fixed holder. Due to the significant absorbance differences at the interface between the irradiated area and unirradiated area, this interface could be easily determined by the position where the absorbance dramatically changed, when the height of the cuvette was adjusted slowly. Subsequently, the absorbances at other positions were obtained with the aforementioned homemade holder.

In order to match with the optical measurements, magnetic resonance imaging (MRI) was also further applied to diffusion measurements. For both the traditional FPX dosimeters and the core-shell FPX@PDMS dosimeters, the MR relaxation rate (1/T_1_) was measured on a clinical 3.0 T GE Discovery 750 MR scanner (General Electric Company, Boston, USA), which was kindly provided by Information Science Laboratory Center of University of Science and technology of China (Hefei, China). M3D/BRAVO/20 sequence was used, and the parameters included echo train length = 4, echo time (TE) = 3.5 ms, repetition time (TR) = 8.6 ms, inversion time (TI) = 50, 100, 200, 300, 400, 600, 800, 1600 or 4000 ms. The MRI images were analyzed by homemade scripts.

### 2.9. Dosimetry of FPX@PDMS Dosimeter

To determine the effect of PDMS coatings on the dosimetry of the FPX@PDMS dosimeter, MCNP software (version 4C), based on the Monto-Carlo method, was used to calculate the dosimetry. During the modeling process, the size of the FPX@PDMS pellets was consistent with the previous experiment, and a parallel ^60^Co photon beam was applied to the dosimeter. F4 tally was used to calculate the average photon fluence normalized to be per starting particle from cell 1 to cell 30 [37], and the relative standard deviation of all calculation results was less than 5%.

## 3. Results and Discussion

### 3.1. Optimization and Characterization of FPX Pellets

**Figure 3:**
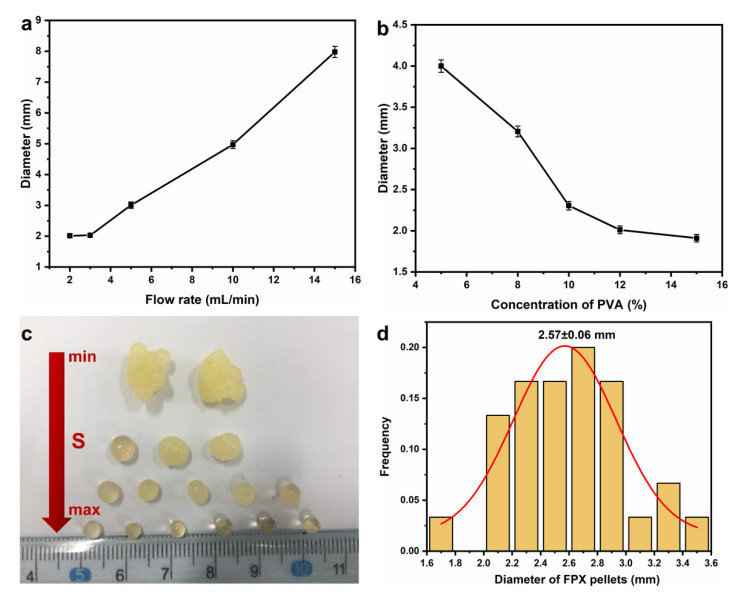
Optimization of preparation parameters of the FPX pellets. (**a**) Diameter of the FPX pellets at different flow rates. (**b**) Diameter of the FPX pellets at different concentrations of PVA. (**c**) Optical photograph of the FPX pellets at different oscillation speeds. (**d**) Size distribution of the FPX pellets.

High-quality FPX pellets are a prerequisite for subsequent dosimeter preparation, and the optimization of preparation parameters related to reducing the diameter and improving the monodispersibility of the FPX pellets is necessary. In general, the quality of the FPX pellets is mainly controlled by four factors: flow rate of the FPX precursor solution (V), concentration of PVA (C) and oscillation speed of micro-oscillator (S). The influence of V on the FPX pellets was shown in Figure 3a. The FPX pellets’ diameter decreased with the decrease in V in the range of 3–15 mL/min, but it hardly changed when V was less than 3 mL/min. This is because FPX droplets are mainly affected by surface tension and gravity, and the decrease in V slows FPX droplet aggregation down at the needle before being frozen. Besides, there is a minimum surface tension in the case of force balance, and therefore the diameter would not always decrease. Furthermore, the FPX pellets’ diameter as a function of C was studied, as shown in Figure 3b. The diameter decreased with the increase in C, and this could be explained by Tate law [38]:

$$diameter={\left(\frac{6\gamma f}{\rho g}\right)}^{1/2}$$

*γ*, *f*, *ρ* and *g* are the surface tension, correction factor, mass density of droplets and acceleration of gravity, respectively. When the concentration of PVA increases, γ would decrease and ρ increases at the same time, and therefore the diameter decreases. However, high-viscosity PVA would fail in droplets formation from the needle, and 10% (*w*/*w*) PVA is preferred. The effect of S on morphology of the FPX pellets was also investigated, as shown in Figure 3c. Adhesion between the pellets occurred and the quality of the pellets was really poor when S was too slow, but high-quality pellets with uniform size would be obtained by increasing S properly. Under optimal conditions, the average diameter of FPX pellets was 2.57 ± 0.06 mm (Figure 3d). Although it might meet the requirement of spatial resolution (<3 mm) of a dosimetric system for medical applications [39], the pellets with a smaller diameter will be needed for developing the high-performance 3D dosimeter. This could be realized by combining with microfluidic technology, improving the hydrophobicity of the inner surface of the needle, reducing the diameter of the needle and using surfactants, etc.

### 3.2. Optimization and Characterization of Core-Shell FPX@PDMS Pellets

**Figure 4:**
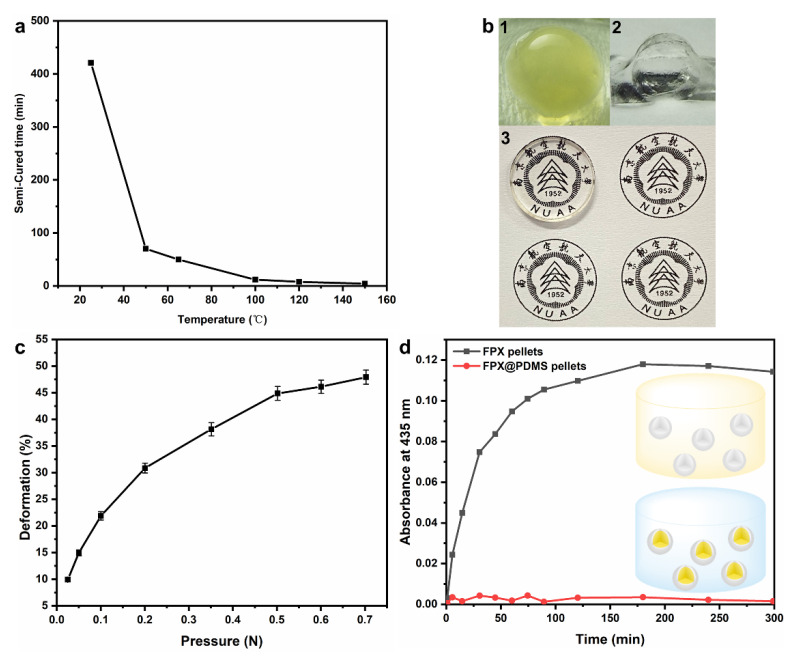
Optimization and characterization of the core-shell FPX@PDMS pellets. (**a**) Semi-cured time of PDMS at different temperatures. (**b**)1 Optical photograph of the FPX@PDMS pellet; 2 Optical photograph of PDMS coating (inner FPX pellet has been removed); 3 Transparency of PDMS coating. (**c**) Deformation of the FPX@PDMS pellets as a function of pressure. (**d**) Absorbance characterization of diffusion of XO from the FPX pellets and the FPX@PDMS pellets.

Rapid and complete preparation of PDMS coatings on the surface of the FPX pellets is vital for fabricating the core-shell FPX@PDMS pellets, and PDMS prepolymer with medium viscosity in a semi-cured state is expected. The effect of temperature on semi-cured time was therefore studied (Figure 4a). Obviously, the semi-cured time was significantly shortened with the increase in temperature. However, high temperature might oxidize Fe^2+^ in FPX pellets, and 50 °C was therefore considered as the optimum temperature; the corresponding semi-cured time was 70 min. The morphology of the FPX@PDMS pellets and PDMS coatings were shown in Figure 4b. A complete and highly transparent PDMS coating with a thickness of 0.4 mm was observed, which indicated the core-shell structure formation; high transparency ensured the accuracy of dose measurement by optical method. The mechanical property of the pellets was shown in Figure 4c, and the maximum deformation was less than 50%. The influence of PDMS coatings on diffusion suppression was shown in Figure 4d, and the FPX pellets without PDMS coatings were the control. Under a steep concentration gradient, XO in the FPX pellets rapidly diffused into the ultrapure water within 200 min. However, XO in the FPX@PDMS did not diffuse out at all, even at 300 min, due to the existence of PDMS coatings. Namely, the diffusion between the pellets was completely suppressed and the diffusion coefficient between the pellets was equal to zero.

### 3.3. Rheological Behavior of PVA Gels

**Figure 5:**
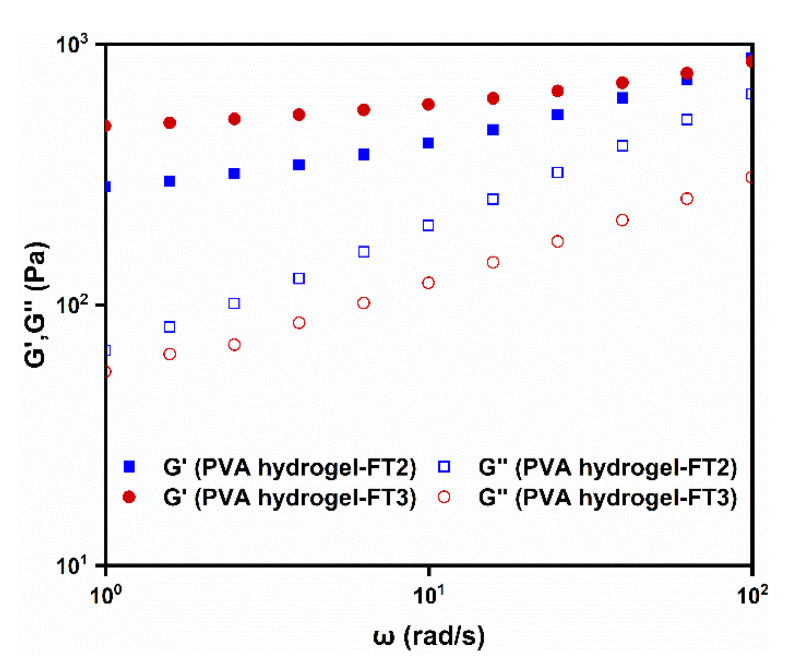
Rheological frequency sweep related to PVA hydrogels with being repetitively frozen and thawed two times (PVA hydrogel-FT2) and three times (PVA hydrogel-FT3).

Figure 5 depicts the frequency sweep results of the PVA hydrogels with different treatments. Within the measured frequency range, the storage modulus G’ were always higher than the loss modulus G″, which proved the solid-like elasticity of the hydrogels characteristic. Furthermore, G’ of the PVA hydrogel-FT3 was greater than those of PVA hydrogel-FT2, which indicated that the PVA hydrogel-FT3 had a greater cross-linking density and compression resistance.

### 3.4. Micro-Mechanical Properties of FPX and FPX@PDMS Pellets

**Figure 6:**
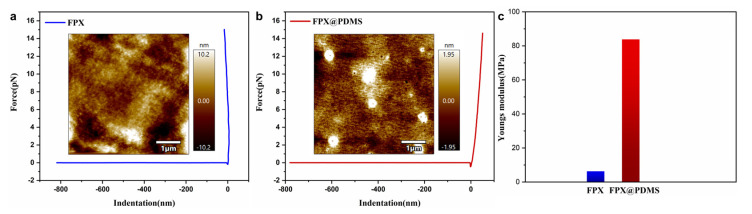
(**a**) Force-distance curves of FPX pellets (inset was the corresponding micro morphology) (**b**) Force-distance curves of FPX@PDMS pellets (inset was the corresponding micro morphology) (**c**) Comparison of Young’s modulus between FPX pellets and FPX@PDMS pellets.

Figure 6a,b show the force-distance curves of the FPX pellets and the FPX@PDMS pellets in AFM force spectroscopy, and the Young’s modulus of FPX pellets and FPX@PDMS pellets were calculated to be 6.30 MPa and 83.83 MPa, respectively, based on Hertz model, as shown in Figure 6c. Due to the existence of the PDMS shell, the Young’s modulus of the core-shell pellets were more than 12 times higher than that of FPX pellets, which indicated that FPX@PDMS pellets had excellent compression resistance properties. This was in good agreement with the result of Figure 4c.

### 3.5. Diffusion Behavior

**Figure 7:**
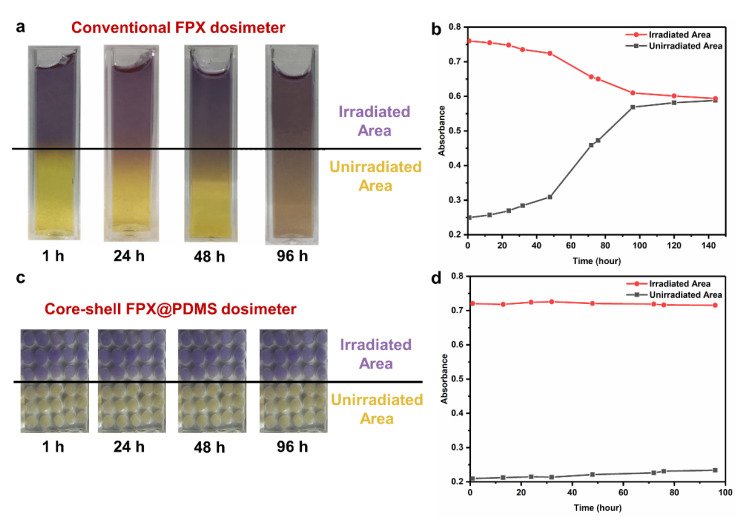
Diffusion comparisons between a conventional FPX dosimeter and a core-shell FPX@PDMS dosimeter. (**a**) Optical photographs of the conventional FPX dosimeter at different times after irradiation. (**b**) Absorbance measurement of diffusion in the conventional FPX dosimeter. (**c**) Optical photograph of the FPX@PDMS dosimeter at different times after irradiation. (**d**) Absorbance measurement of diffusion in the conventional FPX dosimeter.

Diffusion comparisons between conventional the FPX dosimeter and the core-shell FPX@PDMS dosimeter after half-field irradiation were shown in Figure 7. For the conventional dosimeter (Figure 7a), the interface between the irradiated area and the unirradiated area was no longer clear at 24 h after irradiation, and severe diffusion occurred at 96 h after irradiation, and diffusion had been complete at 100 h after irradiation (Figure 7b). However, for the core-shell FPX@PDMS dosimeter, diffusion hardly occurred between the pellets, even at 96 h after irradiation (Figure 7c,d). The results indicated that PDMS coatings effectively suppressed the diffusion of Fe^3+^ in 3D FPX@PDMS pellets and core-shell pellets had a potential in practical 3D dose measurement application.

**Figure 8:**
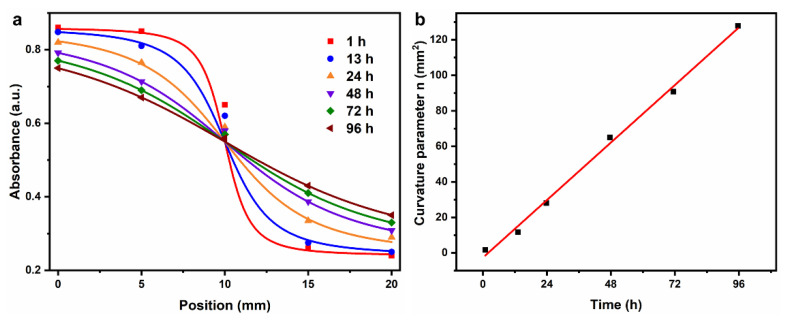
(**a**) Absorbance of Fe^3+^-XO complex of FPX dosimeter at different positions as a function of time after irradiation. (**b**) Linear fitting of curvature parameter to time after irradiation.

Considering the geometric complexity of the target area for radiotherapy, a situation would occur in practical applications: only part of the pellet is irradiated within a single pellet, however, the other part is unirradiated. In other words, although no diffusion was observed between the FPX@PDMS pellets, there might still be ion diffusion inside the pellets. ISQR formula [40] was used to calculate the diffusion coefficient of Fe^3+^ in FPX pellets as a result (Figure 8a), according to the following equation.

$$A\left(x\right)=A_{min}+\frac{1}{2}\left(A_{max}-A_{min}\right)\left[1-\frac{x}{\sqrt{x^{2}+n\left(t\right)}}\right]$$

$A\left(x\right),\;A_{max},\;A_{min},\;n\left(t\right)$ were the absorbances at different positions (*x*, mm), maximum absorbance, minimum absorbance and curvature parameter related to time “*t*” respectively. The diffusion coefficient was determined when dn/dt was multiplied by the factor 0.212 (Figure 8b), and it was calculated to 0.238 mm^2^h^−1^. Besides, the diffusion within the FPX@PDMS pellets could be also corrected according to the mathematical model based on inverse problem techniques proposed by Vedelago et al. [41]. However, a detailed analysis is beyond the scope of this work.

**Figure 9:**
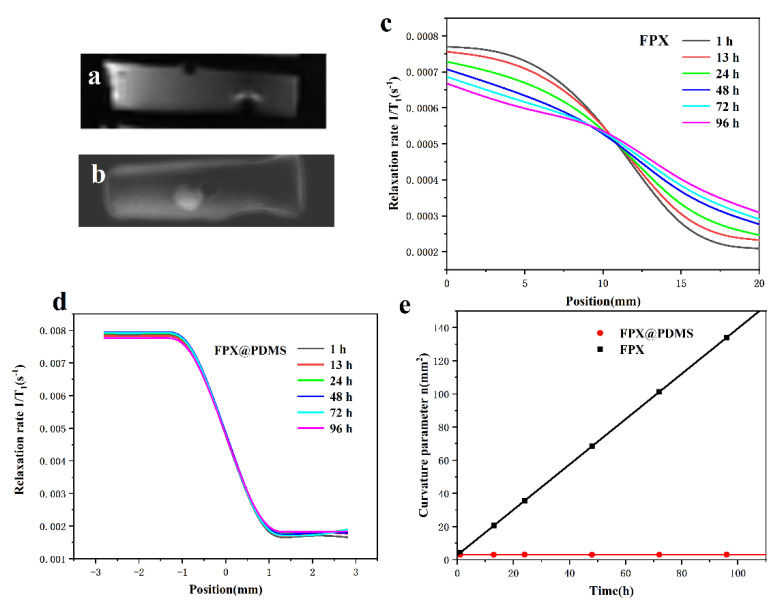
(**a**) MR image of conventional FPX dosimeter used to study the Fe^3+^ diffusion. (**b**) MR image of core-shell FPX@PDMS dosimeter used to study the Fe^3+^ diffusion. (**c**) Relaxation rates of the FPX dosimeter at different positions as a function of time after irradiation. (**d**) Relaxation rates of the FPX@PDMS dosimeter at different positions as a function of time after irradiation. (**e**) Comparisons of linear fittings of curvature parameter to time after irradiation in FPX dosimeter and FPX@PDMS dosimeter.

Figure 9a,b show the MR images of a conventional FPX dosimeter and a core-shell FPX@PDMS dosimeter, respectively. As expected, the MR signal in the area with irradiation (the left side of the image) was more intense, compared to the area without irradiation (the right side of the image). This was ascribed to the fact that radiation-induced Fe^3+^ ions reduced the T_1_ relaxation times and increased the relaxation rates 1/T_1_ and the MR signal in T_1_-weighted images [26]. Figure 9c,d depict the relaxation rates of the FPX dosimeter and the FPX@PDMS dosimeter at different positions as a function of time after irradiation, respectively. It is observed that for the conventional FPX dosimeter, the relaxation rates at different positions obviously changed with the increasing time, which indicated the occurrence of diffusion. However, for the core-shell FPX@PDMS dosimeter, the relaxation rates hardly changed as the time increased, which proved that there was no diffusion between the FPX@PDMS pellets. These results match the aforementioned optical measurements well. Furthermore, to quantitatively obtain the diffusion coefficients, the linear fittings of the curvature parameter to time after irradiation in the FPX dosimeter and the FPX@PDMS dosimeter were performed by the ISQR formula, where the relaxation rates 1/T_1_ were used instead of the absorbance A, as shown in Figure 9e. The diffusion coefficients in the FPX dosimeter and between the FPX@PDMS pellets were calculated as 0.289 mm^2^h^−1^ and 3.25 × 10^−5^ mm^2^h^−1^, respectively. The diffusion coefficient obtained by MRI was larger than that of optical method, which might be attributed to the higher measurement sensitivity of MRI.

### 3.6. Dosimetry of 3D Fricke Gel Dosimeters with Core-Shell Structures

In terms of the interactions of ionizing radiation with materials, the element compositions of dosimeter materials are important, as shown in Table 1. PVA hydrogel substrate and FPX core, as excellent tissue equivalent materials, had similar weight percentages of the element, however, the PDMS shell contained 37.875% silicon (*w*/*w*). Since the dosimetry of FPX dosimeter has been widely studied, we focused our attention on the influence of PDMS coatings on dosimetry of the FPX@PDMS dosimeter.

**Figure 10:**
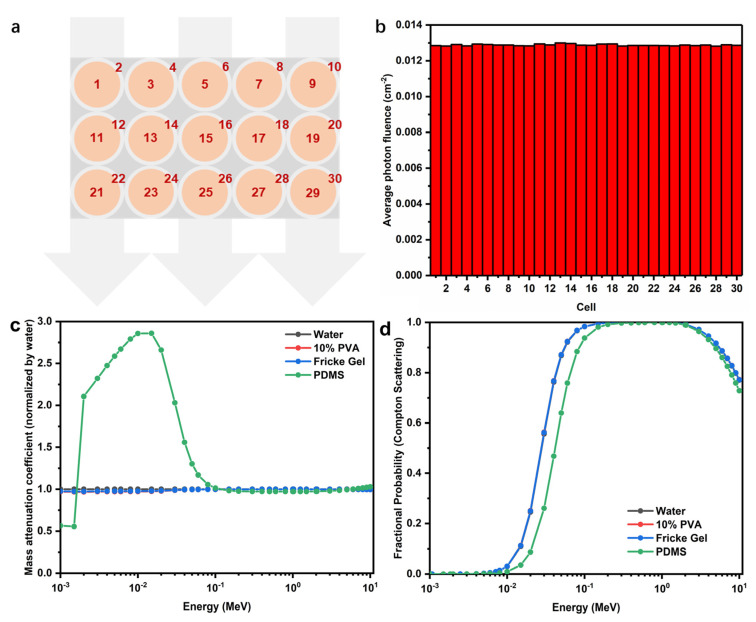
(**a**) Modeling diagram of FPX@PDMS dosimeter. (**b**) Average photon fluence (normalized to per source particle) in different cells. (**c**) Normalized mass attenuation coefficient as a function of photon energy in different materials. (**d**) Fractional probability of Compton scattering as a function of photon energy in different materials.

The normalized average photon fluences in different cells (FPX cores and PDMS shells), based on Monte Carlo calculation, were shown in Figure 10b and the values were really similar. This indicated that PDMS shells hardly changed the dosimetric properties of the FPX@PDMS dosimeter. To illustrate this point, the NIST XCOM database [42], with a mixture rule option over the energy span from 0.001 to 10 MeV, was used to calculate the normalized mass attenuation coefficients of different materials. As shown in Figure 10c, the mass attenuation coefficients between four materials and photons were almost identical in the energy range over 0.1 MeV to 10 MeV, which covered the energy of the photon beam in clinical radiotherapy, and it accorded with Monte Carlo simulation. Compton scattering was the dominant interaction for the MeV photon beams. The fractional probabilities of Compton scattering under different photon energies were shown in Figure 10d, and the probability was close to 1 for therapeutic MeV photon beam for any of the four materials, further confirming the results of Figure 10c. In addition, it should be noted that the curves therefore overlapped with each other because of excellent water equivalence of 10% PVA and Fricke gel.

## 4. Conclusions

In summary, an apparatus and method based on microdroplet, ultrarapid freezing and coating technology for preparing core-shell FPX@PDMS pellets was proposed. The pellets had excellent size (<3 mm), optical transparency and mechanical property, and Fricke gel dosimeters, with core-shell structure based on spatial confinement, were therefore constructed. The Fe^3+^ diffusion was significantly reduced and the diffusion coefficient between the pellets was reduced to almost zero, which was confirmed by both optical method and MRI technique. PDMS coatings did not change the dosimetry within the energy range used in clinical radiotherapy. In future work, the size of the pellets and the thickness of the coatings should be further reduced to achieve the high spatial resolution measurement in practical applications.

## Figures and Tables

**Table 1:** Weight percentage of elements in different materials.

Material	Weight Percentage of Elements (%, *w*/*w*)							
	C	H	O	N	S	Fe	Na	Si
PVA substrate	5.453	10.967	83.508	\	0.072	\	\	\
FPX core	4.545	11.000	84.381	9.338 × 10^−5^	0.074	9.307 × 10^−5^	1.5338 × 10^−5^	\
PDMS shell	32.394	8.156	21.575	\	\	\	\	37.875

## Data Availability

Data sharing is not applicable to this article.

## Abbreviations

PDMS	polydimethylsiloxane
3D	three-dimensional
FPX	Fricke-PVA-xylenol orange
PVA	poly(vinyl alcohol)
XO	xylenol orange
AFM	atomic force microscopy
MRI	magnetic resonance imaging
TE	echo time
TR	repetition time
TI	inversion time
